# General practice management of COPD patients following acute exacerbations: a qualitative study

**DOI:** 10.3399/BJGP.2022.0342

**Published:** 2023-02-21

**Authors:** Bianca Perera, Chris Barton, Christian Osadnik

**Affiliations:** GP, School of Primary and Allied Health Care;; School of Public Health and Preventive Medicine;; Department of Physiotherapy, Monash University, Victoria, Australia.

**Keywords:** COPD, exacerbations, general practice, general practitioners, qualitative research

## Abstract

**Background:**

Exacerbations are the strongest risk factor for future exacerbations for patients living with chronic obstructive pulmonary disease (COPD). The period immediately following exacerbation is a high-risk period for recurrence and hospital admission, and is a critical time to intervene. GPs are ideally positioned to deliver this care.

**Aim:**

To explore perceptions of GPs regarding the care of patients following exacerbations of COPD and to identify factors affecting the provision of evidence-based care.

**Design and setting:**

A descriptive qualitative study was undertaken involving semi-structured, in-depth interviews with Australian GPs who volunteered to participate following a national survey of general practice care for COPD patients following exacerbations.

**Method:**

Interviews were conducted via the Zoom video conference platform, which were audio-recorded and transcribed verbatim. QSR NVivo was used to support data management, coding, and inductive thematic analysis.

**Results:**

Eighteen GPs completed interviews. Six key themes were identified: 1) GPs’ perceptions and knowledge in the management of COPD patients following exacerbation and admission to hospital; 2) pharmacological management; 3) consultation time; 4) communication between healthcare professionals; 5) access to other health services; and 6) patient compliance.

**Conclusion:**

Delivery of post-exacerbation care to COPD patients is affected by GPs, patients, and health service-related factors. The care of COPD patients may be further improved by supporting GPs to overcome identified barriers.

## INTRODUCTION

Acute exacerbations of COPD (AECOPDs) are important events that involve sustained worsening of symptoms that are beyond normal day-to-day variations and necessitate change in regular medication.^1^^–^^3^ Exacerbations are problematic as they are associated with rapid loss of lung function, increased healthcare expenditure related to hospital admissions, and reduced survival.^4^^,^^5^ Exacerbation prevention is therefore a fundamental priority of good COPD management.^6^ Of concern, exacerbations are the strongest risk factor for future exacerbations and become more frequent as the disease progresses.^7^ Recovery from exacerbations is frequently incomplete and may be associated with increased airway inflammation, suggesting it is important to monitor patients after AECOPD.^8^^,^^9^ More than 50% of hospitalised patients are estimated to readmit within 12 months, with the highest risk being the 3 months immediately following discharge.^10^ Therefore, the period immediately after an exacerbation is a critical time to intervene to prevent future exacerbations.

Several hospital-based care models, including clinical pathways and discharge care bundles, have been proposed to improve readmission risk in people with COPD following hospitalisation.^11^^–^^13^ An important issue that is not addressed in the literature is the effective transition of care from acute to community-based primary care. In Australia, where the current study was conducted, the majority of COPD care is delivered by GPs. They can deliver comprehensive post-exacerbation care for COPD patients, provide continuity of care, coordination, and integration of care with other health professionals and services. Evidence-based guidelines, such as Australia and New Zealand’s guidelines for the management of COPD (COPD-X), clearly describe what needs to be actioned following an AECOPD.^14^ Although exacerbations are one of the leading causes of preventable hospital admissions, little evaluation of the provision of care and application of evidence-based guidelines has been undertaken in general practice settings.

## METHOD

### Study design

A qualitative descriptive study was undertaken as part of a larger mixed-methods project of GP management of COPD exacerbations in Australia. The scope of the interviews was informed by the COPD-X guidelines and responses to a nation-wide survey of GPs regarding general practice care of COPD patients following exacerbations.

### Recruitment

Australian GPs who completed the national survey regarding GP care for people following acute exacerbations were invited to participate. This involved purposive distribution of surveys to practising GPs across all Australian states and territories who provided care for patients following AECOPDs in the previous 12 months. At the end of the survey, a convenience sample was established from participants who self-nominated to undertake a subsequent qualitative interview to further explore their personal experiences of care provision for this patient group.

**Table:** How this fits in

The period immediately following an acute exacerbation of COPD is a high-risk period for recurrence and a critical time to intervene. Hospital-initiated, guideline-based care bundles have been previously proposed to optimise post-exacerbation care and reduce readmission; however, convincing evidence of effectiveness has been lacking. As post-exacerbation care is mainly delivered by GPs in Australia, this study describes detailed insights from their perspectives regarding factors affecting the provision of evidence-based care in the period following hospital discharge. The findings highlight factors that should be addressed to enhance care of COPD patients to prevent future exacerbations and hospital readmissions.

### Data collection

In-depth interviews were conducted via the Zoom video conference platform between April and October 2021 by the lead author, who is a practising GP and PhD candidate. Interviews were conducted at a time convenient to the participants. A semi-structured interview guide was developed following the approach of Minichiello *et al*.^15^ This approach involved a recursive model of interviewing, using a combination of closed and open-ended questions to explore GP perceptions on post-exacerbation care of hospitalised COPD patients and factors impacting the provision of evidence-based care in line with guideline recommendations (Box 1). Throughout, the interview participants were encouraged to share their perceptions and experiences of caring for patients following AECOPD. Digital recordings of interviews were transcribed verbatim by a professional transcriptionist service. Field notes and a reflective research journal were kept as part of the audit trail and to support the reflexive thematic analysis.

**Box 1.** An example of open-ended questions asked as part of the semi-structured interview guide

How important do you think it is for COPD patients to have follow-up appointments with their GP after an acute exacerbation? What are the important issues that you would like to address at a follow-up visit following hospitalisation due to an acute exacerbation of COPD? What is your experience with the transition of COPD patients from hospital to community care following acute exacerbation? What challenges do you come across when delivering evidence-based care for these patients? What strategies would you suggest to improve the care of COPD patients following hospitalisation due to an acute exacerbation?

*COPD = chronic obstructive pulmonary disease.*

After the first three interviews, transcripts and interviewer reflections were reviewed by the co-authors to refine the interview guide and provide feedback on interview technique. All participants were reimbursed with a A$250 gift voucher for associated lost clinical time and were given the opportunity to review their unedited transcripts to make clarifications or expand on what they had described in the interview. Repeat interviews were not needed but three participants returned their transcripts with minor changes. Recruitment ended after 18 interviews. At this point, no new concepts were emerging from interviews and the authors considered this to be a sufficient sample size to address the study aims.

### Data analysis

QSR NVivo (Version 12) was used to support data management and coding. Transcripts were read multiple times by the first author prior to coding and thematic analysis using the 6-step approach to reflexive thematic analysis described by Braun *et al* 2019: familiarising with data, generating initial codes, searching for themes, reviewing themes, defining and naming themes, and producing the report.^16^ A preliminary coding scheme was developed by the first author based on an initial review of transcripts. The initial coding scheme was refined following further discussion, questioning, and probing by co-authors. A coding scheme was finalised and the complete set of transcripts was re-coded. A thematic map and charting were used to explore and understand relationships between concepts; they were also used to identify and clarify meaning-based patterns and features across the dataset. Several meetings were held to further refine and define candidate themes to clarify the scope and ‘core’ of each theme prior to final confirmation of themes.

## RESULTS

### Participant characteristics

Twenty-two GPs expressed interest to participate in a qualitative interview, of which one later declined and three did not respond to email or telephone communications. The 18 participants (6 male, 12 female) had varied experience in general practice ranging from 1.5 to 34 years and practised across a representative spread of Australian states and regions (Table 1). Mean interview duration was 32 minutes (range 22 to 46 minutes).

**Table 1.** Participant characteristics

**Variable**	**Number of participants (%)**
**Sex**	
Male	6 (33.3)
Female	12 (67.7)

**Years of experience**	
1–5	7 (38.9)
6–20	6 (33.3)
>20	5 (27.8)

**Location of the practice**	
***States***	
Australian Capital Territory	1 (5.6)
Northern Territory	1 (5.6)
Queensland	3 (16.7)
South Australia	1 (5.6)
Tasmania	7 (38.8)
Victoria	5 (27.7)
***Modified Monash Model****^1^*	
Metropolitan	8 (44.4)
Regional centres	3 (16.7)
Rural towns	6 (33.3)
Remote communities	1 (5.6)

1 *Modified Monash Model: MM1 — metropolitan areas; MM2 — regional centres; MM3–5 — large, medium, and small rural towns; MM6–7 — very remote and remote communities.^17^*

**Figure 1.**
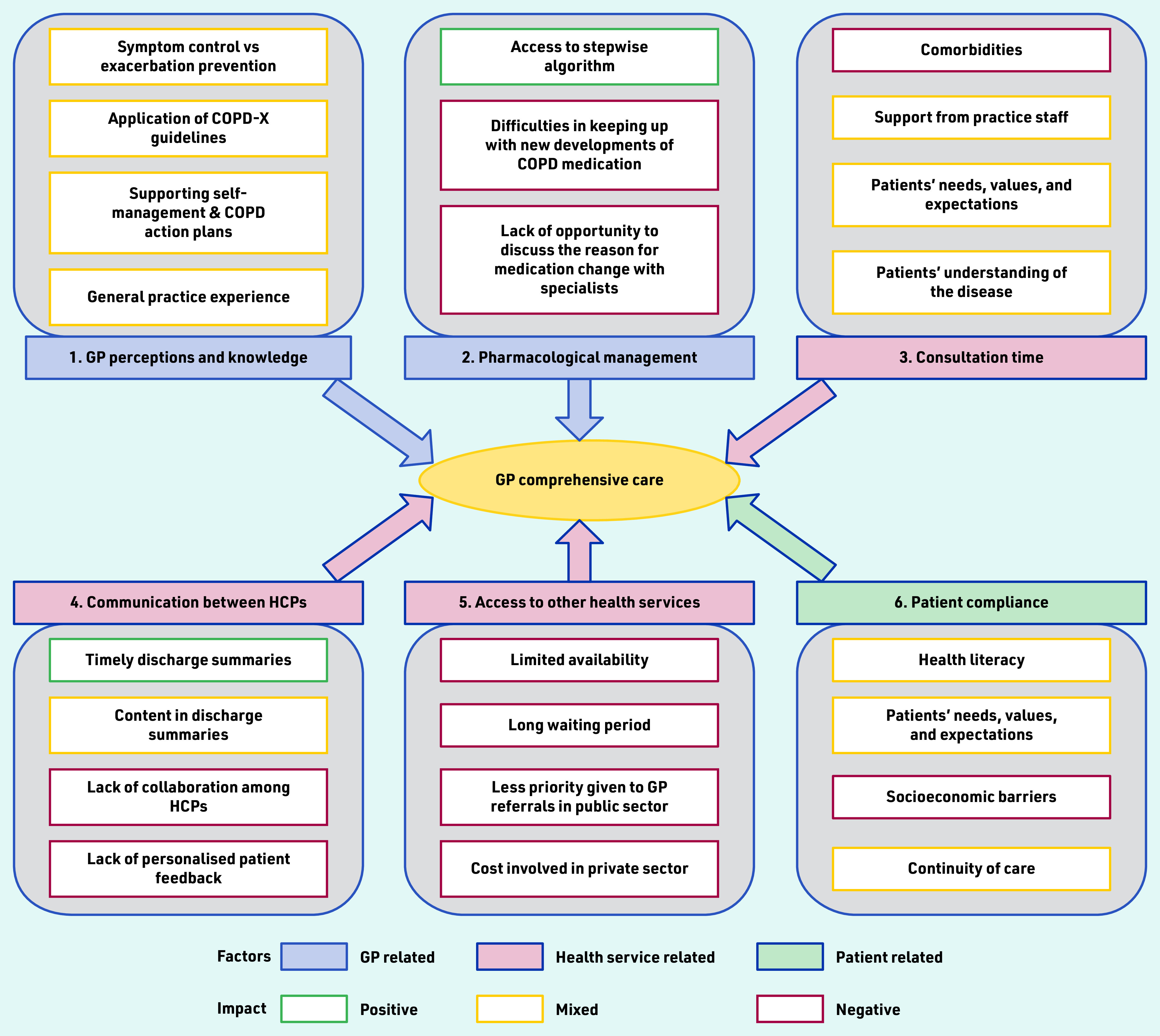
*Factors impacting GP post-exacerbation comprehensive care of COPD patients. COPD = chronic obstructive pulmonary disease. COPD-X = Australia and New Zealand’s guidelines for the management of COPD. HCPs = healthcare professionals.*

Thematic analysis of the interview data identified six key themes that impacted GP care for patients following hospitalisation for AECOPD (Figure 1).

#### Theme 1: GPs’ priorities for Acute Exacerbations of COPD care were varied

All participants considered it important for COPD patients to have a follow-up appointment with their GP after an acute exacerbation, but they differed in terms of perceived priorities and what should happen during this visit and the timing of when it should occur. Some GPs felt the follow-up visit should focus on the patients’ recovery and medication review:

‘*It’s important to help them* [patients] *and see if they’re improving or not and to make sure people are taking the medications and puffers as prescribed.*’

(GP 10, 10 years’ experience)

Others showed a good understanding about exacerbations and considered prevention of exacerbations and readmissions as a priority:

‘*… one exacerbation means, there’s a higher chance of getting another exacerbation.*’

(GP 9, 8 years’ experience)

‘*We have a good COPD management plan in place, including management of further exacerbations, as far as I’m aware it’s best practice to COPD.*’

(GP 12, 10 years’ experience)

GPs also identified the follow-up visit after an AECOPD as an opportunity for the clinician to revisit their management and optimise care to prevent future exacerbations. Furthermore, with long-term doctor–patient relationships, GPs described they were well placed to deliver preventive care:

‘*I think that often the hospital they’re trying to get the patient stable from that exacerbation but really our role is to be taken that as a risk factor for that patient and we know the patient the best, so then try and put in a plan in place to stop further exacerbations*.’

(GP 11, 4 years’ experience)

A small number of GPs in this sample reported using therapeutic guidelines to guide their management of COPD. Among GPs who were aware of Australia’s COPD-X guidelines, perceptions varied. Some felt they were too broad or that it was challenging to find information they needed; others did not find them to be a helpful reference, while others felt the COPD-X ‘concise’ guide^18^ was less complicated and easier to use. More experienced GPs described they were not averse to guidelines, but were more comfortable with their own way of practice. However, they recognised the need for updating their knowledge to ensure practice remained in line with guidelines.

COPD action plans were seen as an important aspect of exacerbation management. Some felt they were very useful to help manage exacerbations and prevent hospital admissions:

‘*Most of my patients are set up with a COPD action plan, I’ve never had a problem, once I’ve educated the patients with them following through, you make sure that the action plan is simple, straightforward, with my patients who’ve got flare ups of COPD, very rarely need to admit them*.’

(GP 8, 26 years’ experience).

In contrast, others viewed action plans more negatively, perceiving that older adults with COPD were not interested in ‘an extra piece of paper’. Some GPs reported that they give verbal advice rather than a written plan. However, GPs commented that participating in the present study had prompted them to consider using written COPD action plans more in their future clinical practice.

Some participants felt barriers such as insufficient consultation time and health literacy restricted their implementation.

#### Theme 2: challenged by pharmacological management of COPD patients

Most GPs reported that they use a stepwise guide for pharmacological management:

‘… *that’s the time I look at the guidelines basically*.’

(GP 9, 8 years’ experience)

Despite the usefulness of the stepwise approach of the Australian COPD-X guidelines, GPs expressed difficulty in selecting the most appropriate medication and inhaler/device as many inhaled medications are available under each category (for example, long-acting muscarinic antagonist, long-acting beta-agonist, and corticosteroids):

‘*I don’t understand them, there are too many, I don’t know the difference between one and another*.’

(GP 12, 10 years’ experience)

Therefore, GPs restricted their selection of medications to the best known, or most familiar to them:

‘*Very overwhelming. I wish there was just one of each, to be honest, I would really. I guess we’ll find our favourites and we just kind of stick with those*.’

(GP 11, 4 years’ experience)

Participants further reflected on their decision making in respect to the medications best suited for patients and the challenges they faced in making these decisions.

Sometimes respiratory physicians commenced new medication, but GPs did not know the reason behind it, feeling it was confusing and complicated. As one GP explained:

‘*Like half the time when they come back from respiratory specialist assessment, they’re on a medication I’ve never heard of. I find it’s complicated and I’m not sure what benefit they give …* [or] *necessarily better than the ones that I am familiar with. So, I find it confusing*.’

(GP 12, 10 years’ experience)

GPs also reported difficulties. They struggled to keep up with new developments in medication and devices used in COPD. Interviews revealed that GPs get to know about medication from advertisements in medical journals or educational sessions sponsored by pharmaceutical companies. They recognised the need to become more knowledgeable in pharmacological management but preferred to have succinct, evidence-based information of COPD medications independent of the pharmaceutical industry:

‘*Keeping up to date with all the new puffers and devices, that’s certainly a challenge*.’

(GP 15, 21 years’ experience)

‘*I found it initially as registrar training very overwhelming, so many drug reps come with all their products and big charts, I just couldn’t get my head around what or which one were better ones*.’

(GP 17, 5 years’ experience)

#### Theme 3: maximising care within constrained consultation time

Follow-up appointments for COPD exacerbations were mostly limited to a standard consultation and limited by the availability of the GP. This varied by practice size and its location. GPs commented on time as a barrier for providing comprehensive review and management advice within a standard consultation. For example, one GP described:

‘*It’s so difficult with the amount of stuff that you have to get through in a 15-minute appointment*.’

(GP 11, 4 years’ experience)

COPD patients also commonly present with comorbidities, therefore the follow-up consultations were not always limited to COPD but also to manage other acute or chronic problems.

Participants felt providing the best possible care at follow-up visit, within the time available for a standard consultation, was a challenge. Addressing their patients’ needs, values, and expectations was a central concern for GPs. One GP explained that:

*‘*… *patients have an agenda and the doctors have an agenda, and often they don’t align. Patient has a list of problems and I have a list of COPD things I want to look through and I can’t do that* [in the time].’

(GP 11, 4 years’ experience)

Many GPs welcomed the role of practice nurses to support the management of patients following AECOPD care, but also highlighted the variability of their role within Australian general practices. Practice nurses were mostly involved with vaccination and spirometry, and some made contributions to patient education, smoking cessation counselling, and checking inhaler technique, but this varied widely.

#### Theme 4: timely communication between healthcare professionals was desired, but experiences were mixed. 

Hospital discharge summaries were identified by GPs as key communication tools.

Most GPs were happy with the timeliness of electronically sent discharge summaries; however, they expressed mixed feelings regarding the content as it showed variation depending on where they worked and the hospital they dealt with. Few GPs were satisfied with the content:

‘*It has improved tremendously over last two or three years, that’s because we get … hospital discharge summaries in our area, which has a message to the GP about what the follow-up plan is and what they want GPs to do, that’s actually very useful.*’

(GP 15, 21 years’ experience)

In contrast, other GPs identified serious concerns with the discharge summaries they had received. It was suggested that the hospital should arrange the follow-up appointment prior to the discharge of the patient:

‘*There are lot of areas they could improve, so to be honest, I find medication aspect is almost useless. Not so much in terms of what happened during the admission, and one of my real frustrations is to follow up, follow-up of the results*.’

(GP 1, >25 years’ experience)

‘*I don’t think the patient generally is told to make an appointment with the GP and it would be really great if the hospital staff could help the patient make a follow-up appointment with the GP before they left the hospital, because it just feels like good handover.*’

(GP10, 10 years’ experience).

Throughout the interviews, GPs described their experiences of communication with outpatient hospital specialists as problematic. One GP described:

‘*The hospital specialists, kind of revise and change things, sometimes without speaking to the community team or GP, then you don’t know, they haven’t spoken to us to understand the reasoning of why they’re on that medication, that’s quite frustrating*.’

(GP 16, 10 years’ experience)

Care providers often have their own disciplinary view of what the patient needs and how they manage the patient. GPs explained the importance of collaboration among healthcare professionals rather than working independently. Interviewees commented that they prefer to have proper feedback from other health professionals who were involved with patient care as it would help to deliver better personalised care for patients. For example, another GP explained:

‘*The correspondence from outpatient pulmonary rehab, a pretty brief summary but I don’t think we get much that’s directly addressing that person as an individual. I prefer it be individualised; it’ll make a world of difference*.’

(GP 15, 21 years’ experience)

#### Theme 5: access to other healthcare services frustrated GPs’ ability to provide best practice care

GPs shared their frustrations of not being able to provide the best practice of care for COPD patients and expressed their concern regarding accessibility of referral services. Less priority given to GP referrals in the public sector, costs involved in private programmes, and limited availability of programmes were identified as barriers to pulmonary rehabilitation:

‘*In terms of the private allied health, that’s quite expensive. So even if people have a team care arrangement and things, you know, at least $50 out of pocket, usually a position that’s just completely out of reach for my patients*.’

(GP 10, 10 years’ experience)

Participants felt that many of these factors were capable of being addressed and, interestingly, felt that referrals for patients following AECOPD might be more appropriately organised by hospital staff before discharge.

Most GP participants also described difficulty accessing outpatient respiratory specialists when required. This included accessibility and affordability issues related to limited availability of respiratory specialists, long waiting period in the public sector, and private sector costs:

‘*It’s very long waiting period to see the specialist, there are only one or two private respiratory specialists here. We’ve got a poor socioeconomic status, so not a lot of people can see them privately. In public hospital, there’s long waiting period to get in.’*

(GP 14, 12 years’ experience)

#### Theme 6: patient compliance with care advice

GPs felt responsible for motivating patients to quit smoking but found it challenging when patients continued to smoke after the provision of support to quit. GPs felt that responsibility for their health ultimately lay with the patients. Some participants described problems regarding discontinuity of care because of patients seeing multiple GPs (as they do not register at a single practice in Australia). This disruption of informational and management continuity was felt to impact on the provision of best care:

‘They’re like, I go to that doctor for that, and I come to you for this, that’s super frustrating.’ (GP 12, 10 years’ experience)

‘*I find it very, very difficult to chase information, I try to explain to patients with ten different doctors, you’re going to get ten different opinions, I’m not saying, and they all have, they have different merits. So, it’s really important to see one person, whoever you like, whoever you trust*.’

(GP 17, 5 years’ experience)

## DISCUSSION

### Summary

This study provides detailed insights from the experiences of GPs responsible for the care of patients following AECOPD in Australia. Post-exacerbation care was influenced by several factors such as GPs’ knowledge and variation in care based on GPs perceived priorities, consultation time, confusion around pharmacological management, problems in communication between healthcare professionals, patient compliance, and difficulties accessing health services. Interviews also revealed key influences that drive these issues related to the GP, patient, and health services.

One of the key concerns to emerge from interviews was uncertainty regarding pharmacological management of COPD. GPs expressed a desire for clearer information to guide inhaler therapy choice at each progression of stepwise management, particularly in light of frequent advances in clinical trials, device types, and combination therapy options. However, it was felt important that such information was received from trusted authorities independent of industry influence. Organisations such as National Prescribing Services have provided such a service in Australia.^19^

Teamwork and communication are crucial elements to deliver effective patient care; however, participants described rarely receiving sufficient clinical information from patients admitted to hospital or engaging in shared decision making with other healthcare professionals. Existing studies of GPs and specialists show areas of shared concerns and communication difficulties among both parties, suggesting a need for ongoing optimisation of feedback exchange.^20^^,^^21^ Involvement of GPs in discharge planning is advocated in COPD-X guidelines and clinical handover to community-based care is an essential standard for hospital staff in Australia’s National Safety and Quality Health Service Standards.^14^^,^^22^ Future audits of compliance with these requirements may be indicated to accurately identify the extent to which this presently occurs.

GPs also described frustrations regarding insufficient time for post-exacerbation consultations to deliver comprehensive care. Initial consultations were typically limited to ‘level B’ (<20 minutes). An enabler proposed by interviewees was a Medicare rebate billing item number for multiple consults (for example, a ‘*COPD cycle of care’*) similar to prior items for asthma and diabetes.^23^^,^^24^ GPs also expressed a desire to strengthen the supportive role of nurses in clinical practice similar to successful models for diabetes educators in primary care for patients following AECOPD.^25^^,^^26^ It remains to be seen whether such initiatives would improve patient care or clinical outcomes.

This study observed variability in the clinical priorities of interviewees that impacted on the application of COPD guidelines. This has also been observed in Australian tertiary hospitals.^27^ As with previous studies, practitioners’ clinical experience and perception were important, but patient needs and expectations were also perceived to influence application of clinical practice guidelines.^28^ Australian general practice is patient centred and places high value on patients’ narratives and shared decision making. This can challenge the delivery of evidence-based care.^29^^,^^30^ Although medical practitioners have a responsibility to maintain knowledge and skills, strategies may need to be considered to increase familiarity with guidelines. Interventions to support the implementation of guideline-based care need to be multifaceted and tailored specifically to the barriers unique to GPs’ health system(s) and funding models within which they operate.^31^ Guidelines are more likely to be used in clinical practice if they are simple, relevant to practice, and perceived as important.^32^ Specific software modules and educational visits have been shown to enhance understanding and implementing guidelines in general practice.^33^

### Strengths and limitations

The qualitative methodology used in this study permitted rich data collection of important insights into general practice care of patients following AECOPD. Participants self-selected (convenience sample) but no participants reported having special interests in respiratory diseases or COPD. This was expected, as recognition of ‘special interest pathways’ is uncommon among Australian GP professions, despite recent creation of such models.^34^ The sample comprised a broad representation of GPs with diverse characteristics such as experience, location, and country where they obtained their medical qualifications. The interviewer’s identity as a GP may also have contributed to the quality and richness of interview data as research has shown professionals interviewing each other (‘communication between equals’) can lead to *‘rich and intuitive responses’*.^35^ However, possible ‘conceptual blindness’ also needs to be considered where interviewer’s feelings and opinion on the subject governs the dialogue.^36^ In this study, experienced qualitative researchers provided oversight of the analysis ensuring rigour and reflexivity.

### Comparison with existing literature

Few participants discussed the role of vaccinations or non-pharmacological management options to potentially prevent future COPD exacerbations. This was interesting as data from the UK show such therapies are highly cost-effective.^37^ Hospital-initiated clinical pathways and AECOPD discharge ‘bundles’ involving delivery of multi-component guideline-based care elements (for example, inhaler technique review, facilitation of smoking cessation, COPD action plans, pulmonary rehabilitation referrals) are documented in the literature and practised in some countries.^11^^–^^13^ Such interventions are, however, rare in Australia. It is therefore unclear whether regional or healthcare organisational differences might explain such observations. Pulmonary rehabilitation is underutilised worldwide for many reasons.^38^ However, barriers identified in this study included referral processes (for example, long wait lists, low acceptance of GP referrals for public programmes), costs (for private programmes), and limited availability of local programmes. Socioeconomic deprivation, poor access to transport, and lack of perceived benefits further contribute to poor uptake rates in previous international studies.^39^^–^^41^ It is interesting that cost emerged as a barrier in this study, considering that many Australian programmes are provided via public health. This may be an artefact of sampling from higher socioeconomic settings; however, it may also suggest a lack of awareness of local public programme availability. Strategies to overcome barriers to undertaking pulmonary rehabilitation continue to require further exploration.

Difficulties prioritising COPD care because of time constraints is a particular pressure point within Australia’s fee-for-service funding model. It has also been highlighted in a Scandinavian study of primary care health professionals.^42^ Time pressures result in clinicians switching reasoning towards intuitive decision-making strategies rather than structured approaches.^43^^,^^44^ This can be counteractive to efforts to enhance evidence-informed practice.^44^ Considering actual time involved and the complexity of the follow-up visit, although level C (long) consultations (>20 minutes, up to 40 minutes) might be more appropriate, evidence is limited to support an association between extending consultation time and quality of care in primary care.^44^^,^^45^

### Implications for research and practice

This study provides valuable insights into Australian GP care of patients following AECOPD. Several discrete issues affecting health service organisation and the delivery of evidence-based medicine were identified and, importantly, appear modifiable. This study highlights a necessity for GPs to maintain familiarity with evidence-based guidelines. This is supported by prior national and international studies with little apparent improvement over time.^46^^–^^50^ Additional support is likely indicated to ensure clinical practice is evidence informed. Much scope remains to explore ways to enhance communication between hospital and primary care settings, including potential engagement of GPs during times of hospital admissions.^51^ Use of GP-informed disease-specific templated clinical handovers and initiatives to strengthen patient and carer engagement regarding discharge planning could potentially further enhance post-exacerbation care. Such opportunities highlight an important ongoing need for future consumer-informed inquiry.
